# Proteome profiling of cerebrospinal fluid using machine learning shows a unique protein signature associated with APOE4 genotype

**DOI:** 10.1111/acel.14439

**Published:** 2024-12-25

**Authors:** Artur Shvetcov, Shannon Thomson, Ann‐Na Cho, Heather M. Wilkins, Joanne H. Reed, Russell H. Swerdlow, David A. Brown, the Alzheimer's Disease Neuroimaging Initiative, Caitlin A. Finney

**Affiliations:** ^1^ Translational Dementia Research Group, Centre for Immunology and Allergy Research Westmead Institute for Medical Research Sydney NSW Australia; ^2^ Department of Psychological Medicine Sydney Children's Hospital Network Sydney NSW Australia; ^3^ Discipline of Psychiatry and Mental Health, School of Clinical Medicine, Faculty of Medicine and Health University of New South Wales Sydney NSW Australia; ^4^ School of Medical Sciences, Faculty of Medicine and Health The University of Sydney Sydney NSW Australia; ^5^ Human Brain Microphysiology Systems Group, School of Biomedical Engineering, Faculty of Engineering The University of Sydney Sydney NSW Australia; ^6^ University of Kansas Alzheimer's Disease Research Centre Kansas City KS USA; ^7^ Department of Biochemistry and Molecular Biology University of Kansas Medical Centre Kansas City KS USA; ^8^ Department of Neurology University of Kansas Medical Centre Kansas City KS USA; ^9^ Autoimmunity and Amyloidosis Research Group, Centre for Immunology and Allergy Research Westmead Institute for Medical Research Sydney NSW Australia; ^10^ Department of Molecular and Integrative Physiology University of Kansas Medical Centre Kansas City KS USA; ^11^ Neuroinflammation Research Group, Centre for Immunology and Allergy Research Westmead Institute for Medical Research Sydney NSW Australia; ^12^ Department of Immunopathology Institute for Clinical Pathology and Medical Research‐New South Wales Health Pathology Sydney NSW Australia; ^13^ Westmead Clinical School, Faculty of Medicine and Health The University of Sydney Sydney NSW Australia

**Keywords:** Alzheimer's disease, APOE4, cerebrospinal fluid, machine learning, proteomics

## Abstract

Proteome changes associated with APOE4 variant carriage that are independent of Alzheimer's disease (AD) pathology and diagnosis are unknown. This study investigated APOE4 proteome changes in people with AD, mild cognitive impairment, and no impairment. Clinical, APOE genotype, and cerebrospinal fluid (CSF) proteome and AD biomarker data was sourced from the Alzheimer's Disease Neuroimaging Initiative (ADNI) database. Proteome profiling was done using supervised machine learning. We found an APOE4‐specific proteome signature that was independent of cognitive diagnosis and AD pathological biomarkers, and increased the risk of progression to cognitive impairment. Proteins were enriched in brain regions including the caudate and cortex and cells including endothelial cells, oligodendrocytes, and astrocytes. Enriched peripheral immune cells included T cells, macrophages, and B cells. APOE4 carriers have a unique CSF proteome signature associated with a strong brain and peripheral immune and inflammatory phenotype that likely underlies APOE4 carriers' vulnerability to cognitive decline and AD as they age.

## Graphical abstract

We sought to identify the cerebrospinal fluid protein (CSF) changes that are associated with the APOE4 genetic variant to better understand the vulnerability of APOE4 carriers to progress to Alzheimer's disease (AD) with age. To do this, we used machine learning and enrichment analyses to analyze 6082 proteins in the CSF of cognitively normal, mild cognitive impairment, and AD individuals with and without an APOE4 allele.

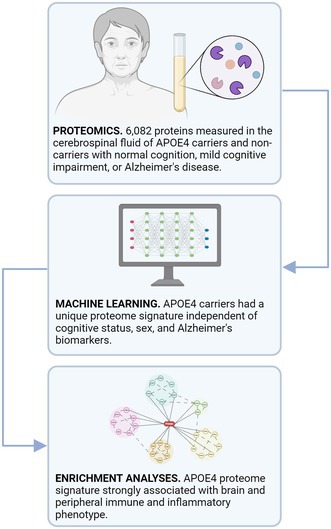

**Abbreviations**
AD: Alzheimer's disease
ADNI: Alzheimer's disease neuroimaging initiative
ANML: adaptive normalization by maximum likelihood
ApoE: Apolipoprotein E
APOE4: Apolipoprotein E ε4
APP: amyloid precursor protein
AUC: area under the curve
BBB: Blood–brain barrier
CART: classification and regression trees
CDR: Clinical Dementia Rating
CSF: cerebrospinal fluid
GO: gene ontology
IHC: immunohistochemistry
MCI: mild cognitive impairment
MMSE: Mini Mental State Examination
NFAT: nuclear factor of activated T cells
NI: non‐impaired
NPV: negative predictive value
PCA: principal component analysis
PCSF: prize‐collecting Steiner Forest
PPI: protein–protein interaction
PPV: positive predictive value
PSEN1: Presenilin 1
PSEN2: Presenilin 2
p‐Tau: phosphorylated tau
RFU: Relative fluorescence units
ROS: reactive oxygen species
t‐Tau: total tau

## 1 BACKGROUND

A variant in the apolipoprotein E gene called ε4 (APOE4) is the single biggest genetic risk factor for late‐onset Alzheimer's disease (AD) accounting for between 40% and 60% of AD's genetic variability (Corder et al., 1993; Safieh et al., 2019). ApoE itself is involved in lipid transport in the plasma and central nervous system and the ApoE4 isoform has been linked to changes in stability that may contribute to protein misfolding (and the misfolded protein response), aggregation, and proteolytic fragmentation (Hatters et al., 2006). APOE4 carriers with AD typically have an onset between 2 and 10 years earlier than their noncarrier counterparts depending on the number of alleles (Corder et al., 1993). Despite the clear relationship between APOE4 and AD, we still have a poor understanding of the underlying mechanisms.

To date, most research has relied on the use of animal models to study the pathomechanisms underlying APOE4 carriage that may have limited translatability to human AD. For example, murine ApoE does not show N‐ and C‐terminal domain interactions (Hatters et al., 2006; Raffai et al., 2001) and there are APOE gene promoter differences between mice and humans (Maloney et al., 2007). Even though murine ApoE can be modified to reproduce some known metabolic properties of human apoE (Hatters et al., 2006; Raffai et al., 2001), or be replaced entirely by human ApoE (Getz & Reardon, 2016), it is not clear how these changes affect the biological system and downstream molecular interactions within it. Further, the use of familial AD mice that express known causal genetic variants in amyloid precursor protein (APP), presenilin 1 (PSEN1) or PSEN2 models only a minority of early onset, familial AD, with unclear implications for modeling the more common late‐onset AD (Drummond & Wisniewski, 2017).

Relative to animal model studies, there are fewer human studies examining potential mechanisms underlying APOE4 carriers' increased AD risk. Previous research has shown that cognitively unimpaired adult APOE4 carriers have changes to cognitive function and memory (Lopez et al., 2018) accompanied by structural and functional brain differences relative to noncarriers. This includes, for example, decreases in hippocampal volume (O'Dwyer et al., 2012), lower neurite density in the entorhinal cortex (Reas et al., 2024), changes in brain co‐activation networks (Filippini et al., 2009; Matura et al., 2014; Sheline et al., 2010), and evidence of higher brain amyloid deposition and tau (Emrani et al., 2020; Hong et al., 2022; Jansen et al., 2015). Human stem cell‐derived astrocytes from APOE4 homozygous carriers were found to be less able to promote neuronal survival and synaptogenesis (Zhao et al., 2017). Stem cell‐derived APOE4 neurons have found enhanced synthesis and intracellular signaling, including via MAP kinase (Huang et al., 2017, 2019), degeneration of GABAergic neurons (Wang, Najm, et al., 2018), higher levels of tau phosphorylation (Wang, Najm, et al., 2018), and increased Aβ production (Huang et al., 2017; Wang, Najm, et al., 2018). Similarly, stem cell‐derived pericytes and blood brain barrier (BBB) models show APOE4 leads to increased amyloid accumulation and dysregulated nuclear factor of activated T cells (NFAT) signaling (Blanchard et al., 2020). Further demonstrating the importance of BBB dysfunction, a recent study found that APOE4 carriers, even those who are cognitively unimpaired, have an increased breakdown of the BBB in the hippocampus and medial temporal lobe linked to pericyte injury and activation of the BBB‐degrading cyclophilin A‐matrix metalloproteinase‐9 pathway (Montagne et al., 2020). In line with a BBB phenotype, recent evidence suggests that APOE4 carriers with AD have differences in cerebrospinal fluid (CSF) and plasma proteins relative to noncarriers with AD (Ayton et al., 2017; Konijnenberg et al., 2020; Miles et al., 2021; Tao et al., 2023). These studies, however, have been limited by including only a small number of proteins (<300) and examining protein changes through the lens of brain‐specific AD pathology including aggregated amyloid‐β. Proteome‐wide changes independent of AD brain pathology and whether these extend to APOE4 carriers irrespective of cognitive status remains unknown, limiting our understanding of whether these CSF proteome changes underly APOE 4 carriers' vulnerability to AD.

To address this, we use a combination of machine learning and functional enrichment analyses to profile the CSF proteome of APOE4 carriers with and without cognitive impairment from the Alzheimer's Disease Neuroimaging Initiative (ADNI) cohort.

## 2 METHODS

### 2.1 Data and participants

We used clinical, APOE genotype, and CSF proteome data generated from the ADNI cohort for this study. All data is accessible through the ADNI database at (https://ida.loni.usc.edu/). A total of 735 participants from the ADNI cohort were identified as either AD, mild cognitive impairment (MCI), or nonimpaired (NI). Diagnostic criteria included Mini‐Mental State Examination (MMSE) scores of 24–30 for NI and MCI patients and 20–26 for AD as well as a Clinical Dementia Rating (CDR) score of 0 for NI, 0.5 for MCI, and 0.5–1 for AD (Petersen et al., 2009). In the current study, participants were allocated to groups (AD, MCI, or NI) based on the diagnosis recorded at their first visit. Participants' age ranged from 71.3 to 76.5 across the groups and included a mix of males and females (Table 1). Clinical progression of cognitive impairment was based on participants' most recent (by year) diagnosis in ADNI3. APOE genotype for ADNI participants was determined by blood sample (Petersen et al., 2009).

**TABLE 1:** Characteristics of the included ADNI cohort participants.

		Ad+	AD−	MCI+	MCI−	NI+	NI−
Total N		209	100	121	162	33	110
Sex (M:F:unknown)		118:90:1	61:39	71:49	91:70:1	20:13	54:56
Age (Average +/− SD)		73.6 ± 0.5	76.5 ± 0.9	71.3 ± 0.7	73.3 ± 0.6	74.5 ± 1.1	74.6 ± 0.6
APOE genotype	e2, e2	—	1	—	1	—	0
	e2, e3	—	7	—	19	—	22
	e3, e3	—	92	—	142	—	88
	e2, e4	4	—	4	—	1	—
	e3, e4	144	—	89	—	30	—
	e4, e4	61	—	28	—	2	—

*Note*: “+” indicates APOE4 carriers and “–” indicates APOE4 non‐carrier.

### 2.2 CSF proteomics

CSF proteome data accessed through the ADNI database was generated by the Neurogenomics and Informatics Centre at Washington University (https://neurogenomics.wustl.edu/) and the Cruchaga Lab at Washington University School of Medicine (https://cruchagalab.wustl.edu/). CSF proteome samples were analyzed using the SomaScan 7 k assay. Data was normalized by SomaLogic including hybridization and median normalization and normalization to a reference using iterative Adaptive Normalization by Maximum Likelihood (ANML) (Wang et al., 2023). Following normalization, additional QC procedures were applied as described in Wang et al., 2023. Protein levels are reported as Relative Fluorescence Unit (RFU). Although there was proteome data for 758 participants from the ADNI cohort in this dataset, APOE genotype was missing for 23 of these and were thus excluded from the current study (final *n* = 735). Protein hits in the CSF proteome file were mapped to Uniprot IDs using the package “SomaScan.db” in R (v4.3.1). Proteins that were unable to be mapped to Uniprot IDs were removed prior to further analysis.

### 2.3 CSF AD pathology burden

To determine whether APOE4 carriers had a high level of AD pathology burden, we identified AD biomarker CSF data including Aβ_42_, total tau (t‐tau), and phospho‐tau181 (p‐tau181). Data was similarly sourced through ADNI and generated by the Department of Pathology & Laboratory Medicine and Centre for Neurodegenerative Diseases Research at the University of Pennsylvania. As described elsewhere (Shaw et al., 2011), Roche Elecsys immunoassays were used to detect Aβ_42_, t‐tau, and p‐tau181 in participant CSF samples according to the manufacturer's instructions. Reference ranges for each analyte were (lower to upper limit) 200–1700 pg/mL for Aβ_42_, 80–1300 pg/mL for t‐tau, and 8–120 pg/mL for p‐tau181 (Shaw et al., 2011).

### 2.4 Statistical analyses

To perform CSF proteome profiling and identify proteins that may be driving between group differences, we employed our unique machine learning methods, as previously described (Finney et al., 2022; Shvetcov et al., 2023). Here, we first use weight‐of‐evidence and information value for feature selection to identify a subset of proteins that are the strongest predictors (>0.3) of group differentiation. We then evaluated the predictive performance of this subset of proteins using classification and regression trees (CART). Importantly, the predictive performance of each protein was evaluated both independently and together with other proteins. This allowed us to identify proteins that may be themselves drivers of between group differences but also proteins that interact together to drive these differences. The CSF proteome dataset was randomly split into 70% training and 30% held‐out testing datasets. CART models were built, fine‐tuned, and validated on the training dataset using five‐fold cross‐validation repeated five times. Models were fine‐tuned using an automatic grid with 100 parameters. An important consideration in the current dataset was the presence of clear class imbalances across the groups (Table 1). More specifically, the n's were unequally distributed between the group which can affect the validity and reliability of machine learning models like CART. To account for class imbalances, we used both oversampling and undersampling techniques to equalize the n's across groups and report model performance metrics that are an average of both. All CART models were evaluated using the 30% held‐out dataset. Performance was determined using several performance metrics including sensitivity, positive predictive value (PPV; also known as precision), specificity, negative predictive value (NPV), and area under the curve (AUC). We also statistically confirmed differences in protein levels between groups using Dunn's Test and Bonferroni adjusted *p*‐values and Wilcoxon effect size calculations.

To identify whether APOE4 carriers were more likely to progress to MCI and AD over time, we used the final recorded diagnosis for each of the 735 participants in the ADNI database. We used a Chi‐squared analysis with *p* < 0.05 to compare the likelihood of progression between APOE4^+^ NI (NI APOE4 carriers) and APOE4^−^ NI (NI APOE4 noncarriers). We also examined whether the APOE4^+^ NI had a high level of AD pathology burden using a linear regression with age as a covariate and *β* and *p* values reported. All data analysis and figures were done in R v 4.3.1 (packages: “Information”, “caret”, “ggplot2”, “FSA”, and “Rstatix”).

### 2.5 Functional enrichment analyses

We examined the relationship between identified proteins that were driving between group differences using protein–protein interaction (PPI) network analyses performed in NetworkAnalyst 3.0 (Zhou et al., 2019). Here, generic PPIs were generated using IMEx interactome data (Breuer et al., 2013). To examine the functional connectivity of the interactive proteins, we applied a Steiner Forest Network analysis that uses a fast heuristic Prize‐collecting Steiner Forest (PCSF) algorithm. We then applied a first‐order network analysis to examine biological and molecular enrichment in Gene Ontology (GO) and Reactome pathways.

For brain region and cell type‐specific enrichment analyses, the Human Protein Atlas was used (Karlsson et al., 2021; Sjostedt et al., 2020) (https://www.proteinatlas.org/) (v23, Ensembl v109). Expression profiles for brain tissue were based on immunohistochemistry using tissue microarrays and data was included for all measured brain regions (cerebral cortex, caudate, hippocampus, and cerebellum) (Sjostedt et al., 2020). Cell type specific data for brain and immune cells were based on single‐cell RNA‐sequencing (Karlsson et al., 2021). For these enrichment analyses, normalized expression was used and heatmaps were generated by using min‐max scaling and in GraphPad Prism (v.10.0.0 for Windows).

## 3 RESULTS

### 3.1 APOE4 carriers do not have increased CSF levels of ApoE protein or its isoforms

**FIGURE 1:**
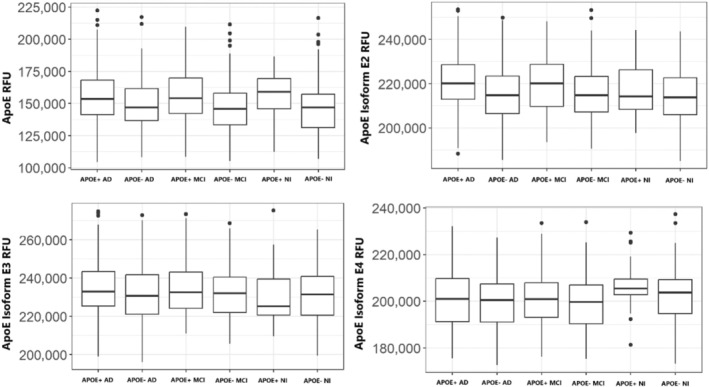
Box plots of the levels of CSF ApoE protein and its isoforms, E2, E3, and E4, across APOE4 carriers of different cognitive statuses. Plots show the relative fluorescent units (RFU) of each. AD, Alzheimer's disease; MCI, Mild cognitive impairment; NI, No impairment.

We first sought to identify whether CSF levels of ApoE protein, and its three isoforms, differed among APOE4 carriers versus noncarriers across NI, MCI, and AD groups. The SomaScan 7 k assay included relative fluorescent units (RFU) for ApoE, ApoE2, ApoE3, and ApoE4. There were no differences in any of the level of these proteins in the CSF across groups, regardless of APOE genotype or cognitive status (Figure 1).

### 3.2 APOE4 carriers have a unique proteome signature irrespective of cognitive status

**FIGURE 2:**
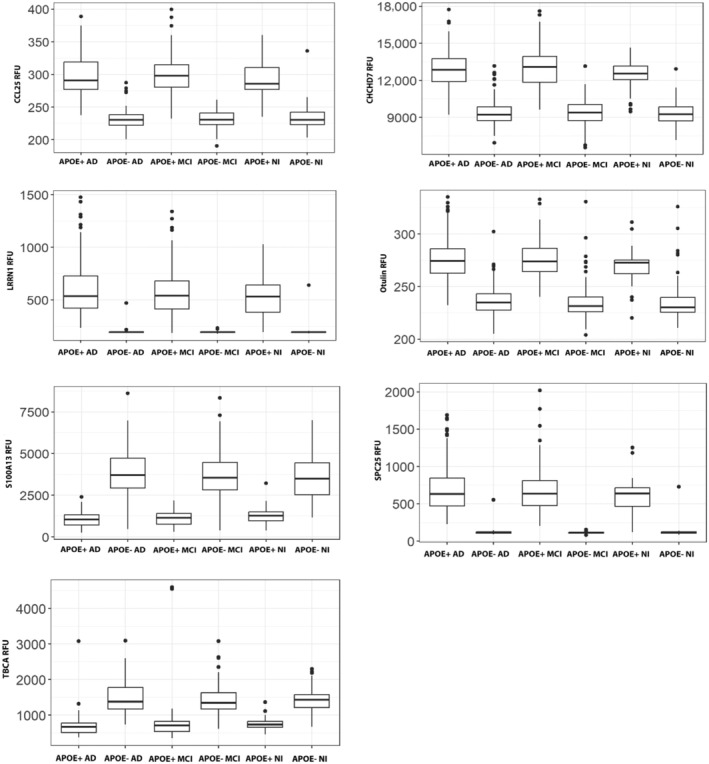
Box plots of the seven independent proteins that each predict APOE4 carriers irrespective of cognitive status. Plots show the relative fluorescent units (RFU) of each protein CCL25, CHCHD7, LRRN1, Otulin, S100A13, SPC25, and TBCA and highlights differences between APOE4 carriers and noncarriers. AD, Alzheimer's disease; MCI, Mild cognitive impairment; NI, No impairment.

We first sought to identify differences in the CSF proteome of APOE4 carriers with AD (APOE4^+^ AD) relative to noncarriers with AD (APOE4^−^ AD). To do this, we performed an initial feature selection of the 6082 proteins identified by the semitargeted proteomics SomaScan 7 k assay using weight‐of‐evidence and information value. This identified 1534 proteins that had strong predictive power (>0.3) of between group differences (Table S1). We then examined the ability of the 1534 proteins, both independently and together, to predict APOE4^+^ AD relative to APOE4^−^ AD using CART. Two groups of proteins had a strong predictive performance (sensitivity >0.75). The first group was characterized by seven “stand‐alone” proteins that each independently predicted APOE4^+^ AD and APOE4^−^ AD cases. Performance metrics for each of the seven independent proteins were all 1.0 for sensitivity, specificity, PPV, NPV, and AUC, respectively. The independent proteins included CCL25, CHCHD7, LRRN1, Otulin, S100A13, SPC25, and TBCA (Table S2). Box plots of these seven independently predictive proteins showed clear, statistically significant separation between APOE genotypes, irrespective of cognitive status, with minimal to no overlap (Figure 2; Table S3). This observation was further supported by large Wilcoxon effect size calculations (Table S3).

The second group of proteins was characterized by 50 proteins that interacted together to predict APOE4^+^ AD and APOE4^−^ AD (interactive proteins; Table S4). These proteins similarly showed high CART performance metrics (sensitivity = 0.99, specificity = 0.74, PPV = 0.86, NPV = 0.98, AUC = 0.92), indicating that they were strong predictors.

We next examined whether this proteome signature of independent and interactive proteins was unique to APOE4^+^ AD or whether it was generalizable to other groups. To do this, we first tested whether our CART models and signatures could generalize to APOE4 carriers with MCI or NI (APOE4^+^ MCI or APOE4^+^ NI). CART models using either the independent or interactive proteins as predictors performed poorly and lost their predictive power (Table 2). We used CART to further test whether our proteins could differentiate between APOE4^+^ MCI and APOE4^+^ NI; similarly finding that our models were unable to do so (Table 2). This demonstrated that both the independent and interactive proteins are the same across all APOE4 carriers, independent of cognitive status (AD, MCI, or NI).

**TABLE 2:** Performance metrics of CART models testing the uniqueness of the identified proteome signature to APOE4 carriers.

	Sensitivity	Specificity	PPV	NPV	AUC
APOE4^+^ AD vs. APOE4^+^ MCI					
Independent Proteins	0.67	0.44	0.78	0.28	0.47
Interactive Proteins	0.67	0.38	0.69	0.37	0.53
APOE4^+^ AD vs. APOE4^+^ no impairment					
Independent Proteins	0.84	0.15	0.47	0.50	0.54
Interactive Proteins	0.88	0.28	0.65	0.50	0.49
APOE4^+^ MCI vs. APOE4^+^ no impairment					
Independent Proteins	0.83	0.13	0.62	0.34	0.53
Interactive Proteins	0.85	0.22	0.74	0.34	0.47
APOE4^+^ MCI vs. APOE4^−^ MCI					
Independent Proteins	1.00	1.00	1.00	1.00	1.00
Interactive Proteins	0.91	0.94	0.93	0.92	0.95
APOE4^+^ no impairment vs. APOE4^−^ no impairment					
Independent Proteins	0.78	0.97	0.90	0.93	0.91
Interactive Proteins	0.73	0.94	0.75	0.93	0.84

**FIGURE 3:**
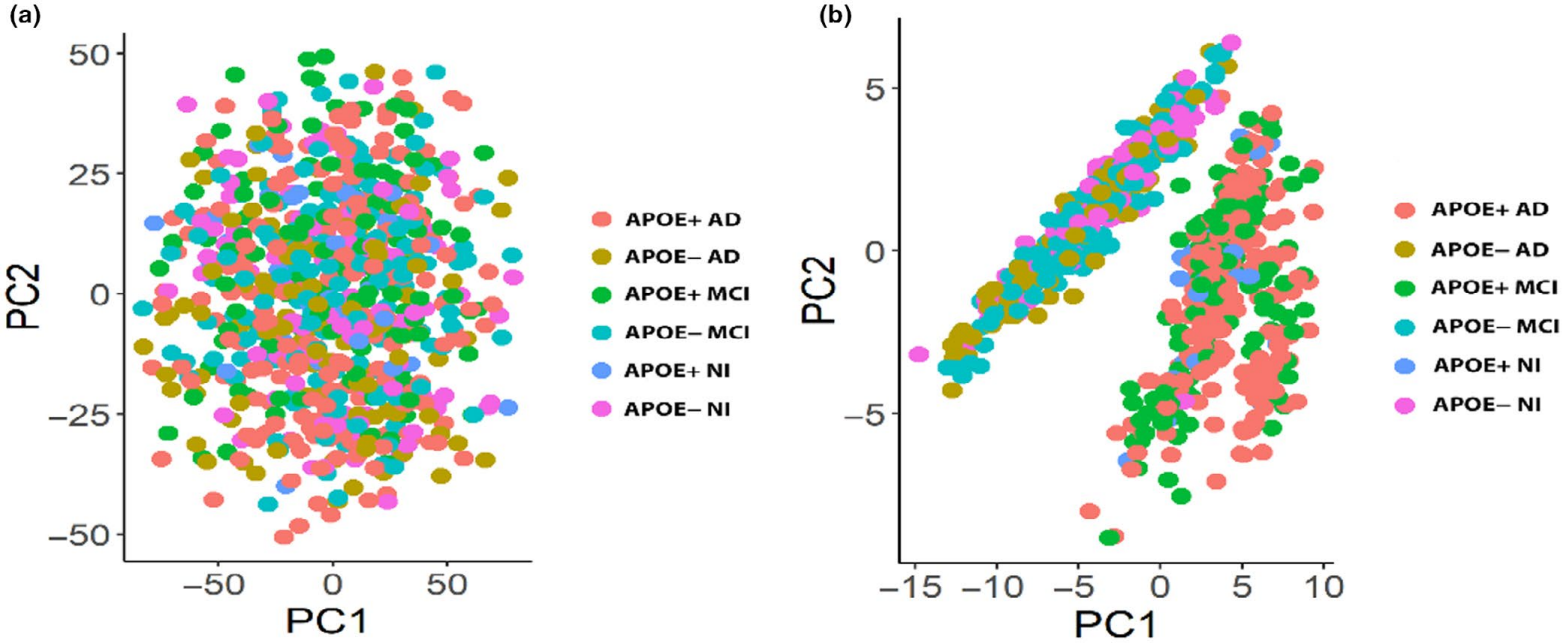
Principal component analysis (PCA) of CSF proteins across experimental groups. (a) PCA of all 6082 proteins included in the present study shows no clear group separation based on cognitive status or APOE4 genotype. (b) PCA of the 57‐protein proteome signature (including both independent and interactive proteins) identified as being unique to APOE4 carriers independent of cognitive status. AD, Alzheimer's disease; MCI, Mild cognitive impairment; NI, No impairment.

**FIGURE 4:**
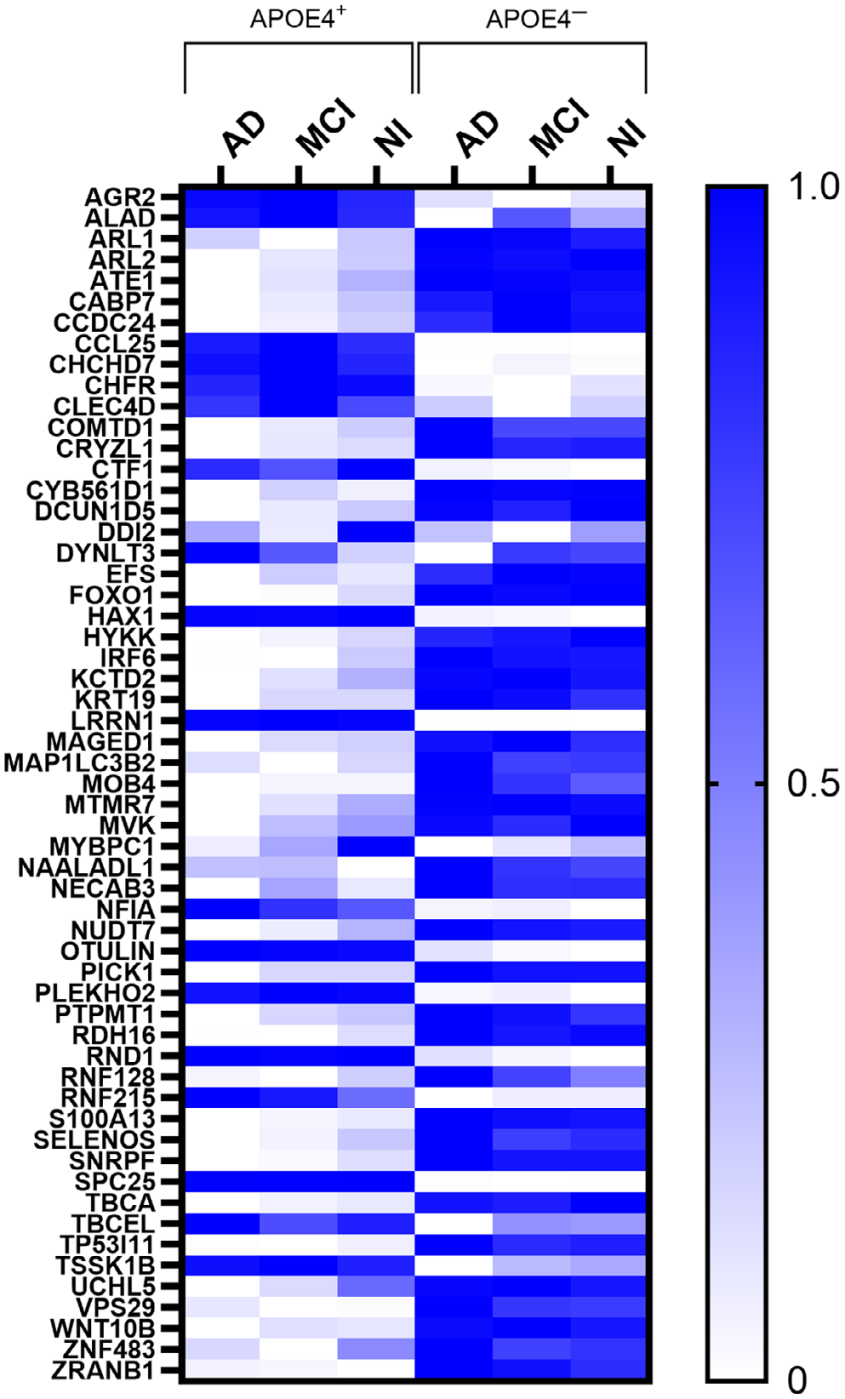
Heat map of the relative levels of 57 proteins (both independent and interactive) identified by CART as being unique to APOE4 carriers relative to noncarriers irrespective of cognitive status. AD, Alzheimer's disease; MCI, Mild cognitive impairment; NI, No impairment; Proteins: AGR2, Anterior gradient protein 2 homolog; ALAD, δ‐aminolevulinic acid dehydratase; ARL1, ADP‐ribosylation factor‐like protein 1; ARL2, ADP‐ribosylation factor‐like protein 2; ATE1, Arginyl‐tRNA‐protein transferase 1; CABP7, Calcium‐binding protein 7; CCDC24, Coiled‐coil domain‐containing protein 24; CCL25, CC motif chemokine ligand 25; CHCHD7, Coiled‐coil‐helix‐coiled‐coil‐helix domain‐containing protein 7; CHFR, E3 ubiquitin‐protein ligase CHFR; CLEC4D, C‐type lectin domain family 4 member D; COMTD1, Catechol O‐methyltransferase domain‐containing protein 1; CRYZL1, Quinone oxidoreductase‐like protein 1; CTF1, Cardiotrophin‐1; CYB561D1, Probable transmembrane reductase CYB561D1; DCUN1D5, DCN1‐like protein 5; DDI2, Protein DDI1 homolog 2; DYNLT3, Dynein light chain Tctex‐type 3; EFS, Embryonal Fyn‐associated substrate; FOXO1, Forkhead box protein O1; HAX1, HCLS1‐associated protein X‐1; HYKK, Hydroxylysine kinase; IRF6, Interferon regulatory factor 6; KCTD2, BTB/POZ domain‐containing protein KCTD2; KRT19, Keratin, type I cytoskeletal 19; LRRN1, Leucine‐rich repeat neuronal protein 1; MAGED1, Melanoma‐associated antigen D1; MAP1LC3B2, Microtubule‐associated proteins 1A/1B light chain 3 *β* 2; MOB4, MOB‐like protein phocein; MTMR7, Myotubularin‐related protein 7; MVK, Mevalonate kinase; MYBPC1, Myosin‐binding protein C slow‐type; NAALADL1, Aminopeptidase NAALADL1; NECAB3, N‐terminal EF‐hand calcium‐binding protein 3; NFIA, Nuclear factor 1 A‐type; NUDT7, Peroxisomal coenzyme A diphosphatase NUDT7; OTULIN, Otulin; PICK1, PRKCA‐binding protein; PLEKHO2, Pleckstrin homology domain‐containing family O member; PTPMT1, Phosphatidylglycerophosphatase and protein‐tyrosine phosphatase 1; RDH16, Retinol dehydrogenase 16; RNF128, E3 ubiquitin‐protein ligase RNF128; RNF215, RING finger protein 215; S100A13, S100‐A13; SELENOS, Selenoprotein S; SNRPF, Small nuclear ribonucleoprotein F; SPC25, Kinetochore protein spc25; TBCA, Tubulin‐specific chaperone A; TBCEL, Tubulin‐specific chaperone cofactor E‐like protein; TP53I11, Tumor protein p52‐inducible protein 11; TSSK1B, Testis‐specific serine/threonine‐protein kinase 1; UCHL5, Ubiquitin carboxyl‐terminal hydrolase isoenzyme L5; VPS29, Vacuolar protein sorting‐associated protein 29; WNT10B, Protein Wnt‐10b; ZNF483, Zinc finger protein 483; ZRANB1, Ubiquitin thioesterase ZRANB1.

We then tested whether the proteome signatures were able to differentiate between APOE4 carriers and noncarriers. CART models comparing APOE4^+^ MCI and APOE4^−^ MCI as well as APOE4^+^ NI to APOE4^+^ NI all had a high level of performance, similar to our initial models comparing APOE4^+^ AD to APOE4^−^ AD (Table 2). This indicated that the proteome signature is indeed specific to APOE4 carriers and, to further validate this, we performed a principal component analysis (PCA). This showed that there was no group separation when looking at all 6082 identified CSF proteins (Figure 3a) but very clear group separation based on APOE4 status using our 57‐protein (independent and interactive) proteome signature (Figure 3b). This effect was also visualized using a heat map based on measured protein levels (Figure 4), further highlighting that the CSF proteome signature was indeed unique to APOE4 carriers irrespective of cognitive status.

Given that the proteome signature was specific to APOE4 carriers independently of cognitive status, we then examined whether there were any potential sex differences between all (diagnosis‐independent) male and female APOE4 carriers and noncarriers. To do this, we tested whether our CART models of independent or interactive proteins differentiated males and females. When we compared APOE4^+^ females to APOE4^+^ males, our CART models did not perform well, indicating that these groups were indistinguishable (Table 3). We found the same when we compared APOE4^−^ females to APOE4^−^ males (Table 3). As a further confirmation that our CART models still differentiate between APOE4 carriers and noncarriers, we also tested APOE4+ females versus APOE4‐ males and found that our models again had high performance metrics (Table 3).

**TABLE 3:** Performance metrics of CART models testing the sex differences in the APOE4 protein signature.

	Sensitivity	Specificity	PPV	NPV	AUC
APOE4^+^ Female vs. APOE4^+^ Male					
Independent Proteins	0.55	0.68	0.63	0.61	0.63
Interactive Proteins	0.43	0.57	0.37	0.63	0.49
APOE4^−^ Female vs. APOE4^−^ Male					
Independent Proteins	0.56	0.60	0.29	0.83	0.44
Interactive Proteins	0.61	0.66	0.49	0.76	0.37
APOE4^+^ Female vs. APOE4^−^ Male					
Independent Proteins	1.0	1.0	1.0	1.0	1.0
Interactive Proteins	0.90	0.94	0.93	0.90	0.98

### 3.3 The APOE4 proteome signature is independent of CSF AD pathology burden in nonimpaired controls

Previous research has shown that APOE4 carriers may have a higher AD pathology burden relative to noncarriers, including, for example, higher cortical Aβ deposition (Fouquet et al., 2014) and increased tau spreading (Steward et al., 2023). An important consideration, therefore, was whether our APOE4‐specific proteome signature was a consequence of increased AD pathology burden or occurs independently of this. To determine this, we compared CSF AD biomarker data for Aβ_42_, t‐tau and p‐tau181 for APOE4^+^ and APOE4^−^ NI participants using a linear regression. We also included age as a co‐variate due to research showing that Aβ_42_ and tau levels also increase with normal aging (Blomberg et al., 2001; Rodrigue et al., 2009, 2012; Ziontz et al., 2019) and that the relationship between APOE genotype and AD biomarkers is modified by age (Kester et al., 2009).

Our analysis revealed nonsignificant main effects of APOE4 genotype on t‐tau (*β* = −0.186, *p* = 0.795), p‐tau181 (*β* = −0.166, *p* = 0.831), and Aβ_42_ (*β* = 0.158, *p* = 0.885). Additionally, there was no significant interactions between APOE4 genotype and age for t‐tau (*β* = 0.005, *p* = 0.602), p‐tau181 (*β* = 0.005, *p* = 0.594), or Aβ_42_ (*β* = −0.007, *p* = 0.625), indicating that the relationship between age and CSF AD biomarkers does not differ based on APOE genotype in NI participants. There was, however, significant main effects of age on t‐tau (*β* = 0.013, *p* = 0.005) and p‐tau181 (*β* = 0.015, *p* = 0.003), suggesting that t‐tau and p‐tau181 vary with age regardless of APOE genotype. Unlike tau, we did not find a significant main effect of age on Aβ_42_ levels (*β* = −0.006, *p* = 0.423).

### 3.4 APOE4 carriers are more likely to progress to cognitive impairment and AD

We next sought to identify whether the APOE4 proteome signature was pathogenic or benign by examining whether APOE4 carriers were more likely to progress to clinically diagnosed cognitive impairment over time. We found that APOE4^+^ NI or APOE4^+^ MCI participants in the current study were more likely to progress to either MCI or AD relative to participants without an APOE4 allele (14% vs. 5% progressed, respectively; *X* ^2^ = 7.14, df = 1, *p* = 0.008).

### 3.5 Functional characterization of the APOE4 proteome signature

**FIGURE 5:**
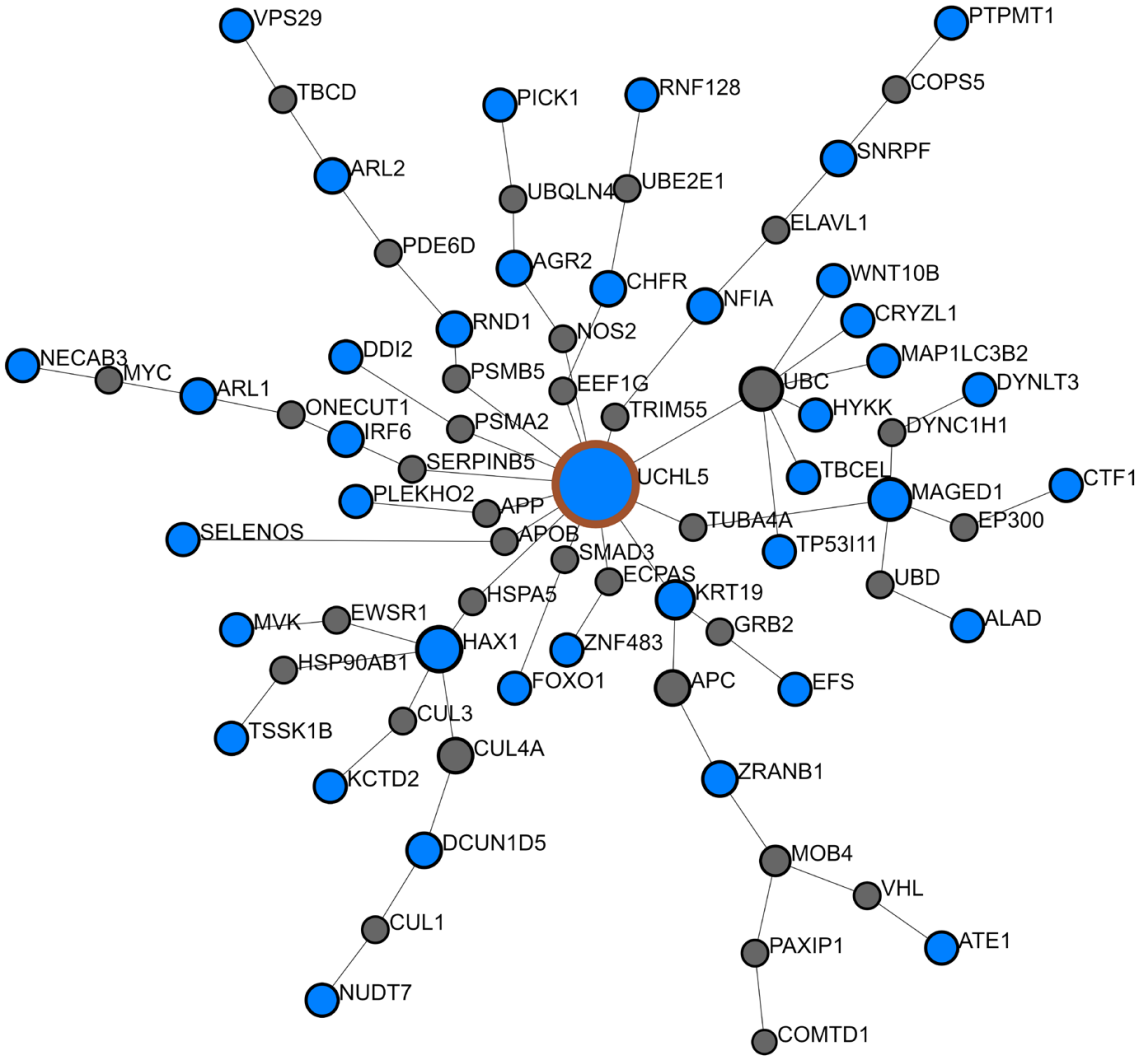
Steiner Forest Network protein–protein interaction (PPI) analysis showing functional connectivity between the interactive proteins (total *n* = 41/50) in the APOE4 proteome signature. Blue nodes indicate proteins identified. Gray nodes indicate inferred proteins.

Finally, we sought to identify potential mechanisms underlying APOE4 carriers' vulnerability to progression. Independent proteins (*n* = 7) within the APOE4 CSF proteome signature were involved in T cell development, synapse assembly, innate immune responses, and cell division (Table S4). We then performed a functional enrichment analysis on the interactive proteins (*n* = 50) because they were interacting together to predict between APOE4 carriers and noncarriers. A Steiner Forest Network protein–protein interaction (PPI) analysis showed that 41/50 proteins formed a clear functional network (Figure 5).

Further pathway enrichment analyses using GO indicated significant involvement in biological and molecular functions including catabolism, protein ubiquitination, mitosis, DNA damage response, ATP binding, and oxidative and cellular stress responses (all adj. *p* < 0.01; Tables S5 and S6). Enriched Reactome signaling pathways included regulation of mitosis, immune system, apoptosis, inflammation and RNA and DNA regulation (all adj. *p* < 0.001; Table S7).

**FIGURE 6:**
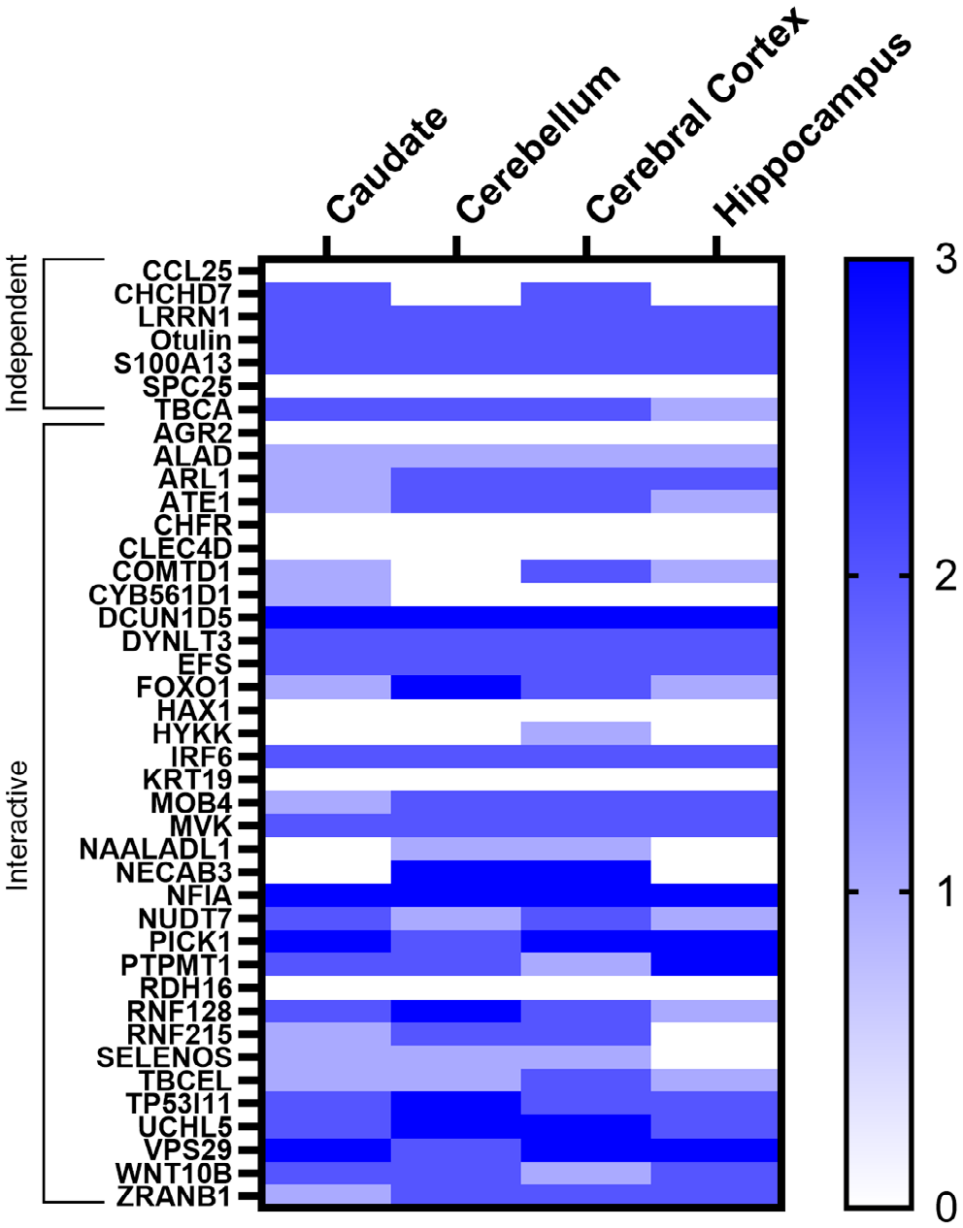
Enrichment across brain regions for the independent (*n* = 7/7) and interactive (*n* = 34/50) proteins within the APOE4 proteome signature based on confirmed immunohistochemistry microarrays from normal tissue in the Human Protein Atlas (Sjostedt et al., 2020).

To determine where our APOE4 proteome signature was enriched in the brain, we performed an enrichment analysis using immunohistochemistry microarray data from the Human Proteome Atlas (Sjostedt et al., 2020). All 7 of the independent proteins were represented in the IHC microarrays however only 34/50 of the interactive proteins were represented. We found our proteome signature, including both independent and interactive proteins, was enriched in the caudate and cerebral cortex and, to a lesser degree, in the cerebellum (Figure 6). Interestingly, proteins within the signature were not especially enriched in the hippocampus (Figure 6).

**FIGURE 7:**
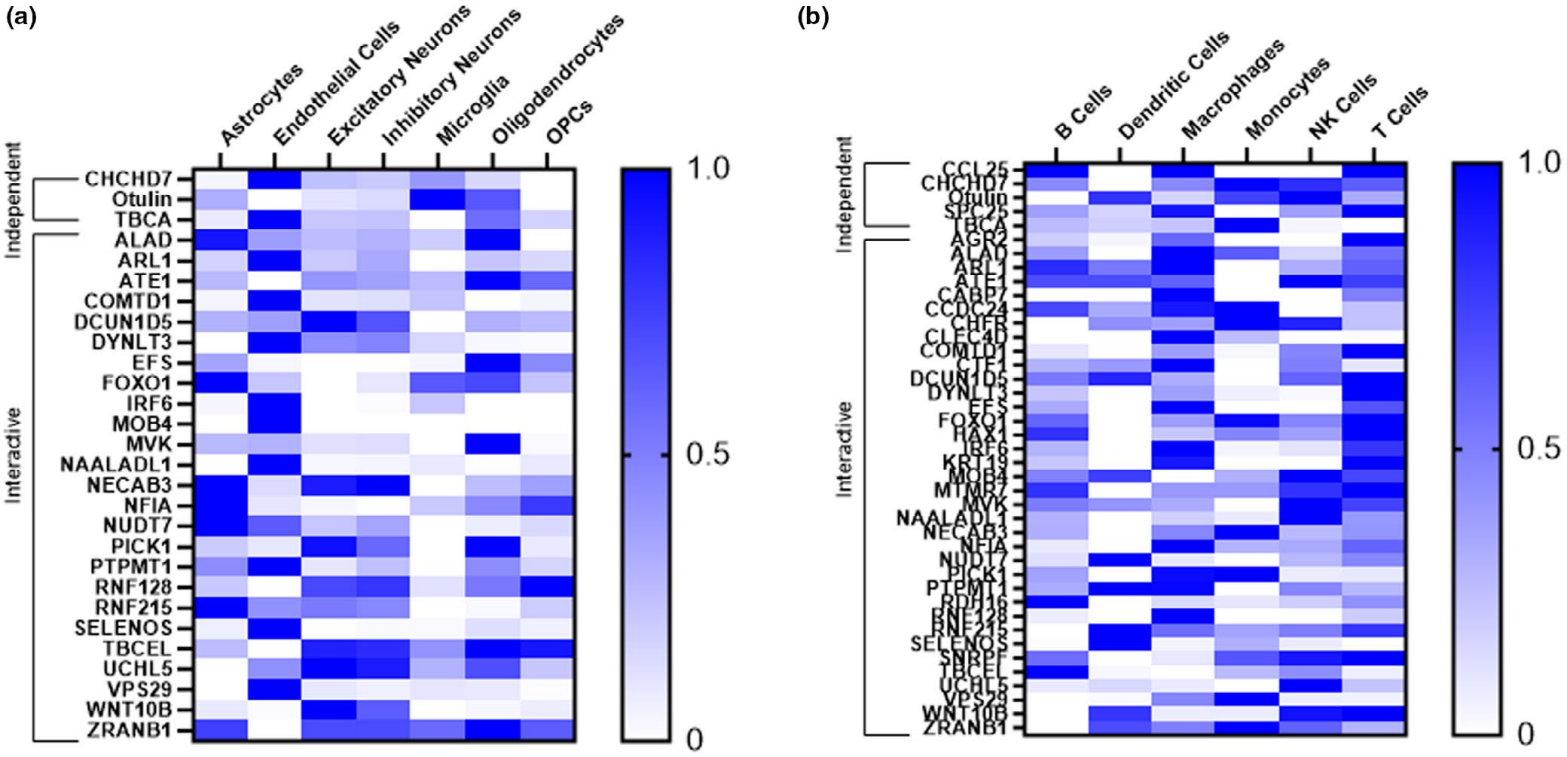
Cell type specific enrichment analyses for proteins within our APOE4 proteome signature across the brain and the periphery using single cell RNA‐sequencing data from normal tissue from the Human Protein Atlas (Karlsson et al., 2021). (a) Enrichment for brain cell subtypes for the independent (*n* = 3/7) and interactive (*n* = 25/50) proteins shown to be expressed in brain tissue by IHC microarrays. (b) Enrichment across peripheral immune cell subtypes for independent (*n* = 5/7) and interactive (*n* = 36/50) proteins. OPC, Oligodendrocyte precursor cells.

We then investigated cell type specific enrichment patterns for proteins within our APOE4 signature using single‐cell RNA‐sequencing data from the Human Protein Atlas (Karlsson et al., 2021). In the brain, and limiting our analysis to only those proteins identified in as being expressed in the brain by IHC microarrays (Figure 6), we found the highest level of enrichment for both independent and interactive proteins in endothelial cells (Figure 7a). Astrocytes and oligodendrocytes were also highly enriched for proteins within our APOE4 proteome signature, a finding in line with the immune and inflammatory pathways that these proteins are enriched for. Interestingly, this central immune dysregulation was also mirrored in the periphery. Single‐cell RNA‐sequencing enrichment analysis for immune cell subtypes indicated that our APOE4 proteins (data found only for *n* = 5/7 independent and *n* = 36/50 interactive proteins) were especially enriched in macrophages and T cells and, to a slightly lesser extent, B cells (Figure 7b).

## 4 DISCUSSION

While unique CSF and plasma proteins have previously been associated with APOE4^+^ AD patients (Ayton et al., 2017; Konijnenberg et al., 2020; Tao et al., 2023), we expand on this existing literature by integrating machine learning and functional enrichment analyses to show that APOE4 carriers indeed have a unique CSF proteome signature consisting of seven independent and 50 interactive proteins. This signature was independent of cognitive status, as it was able to differentiate APOE4 carriers and noncarriers irrespective of whether they were not impaired, MCI, or AD. The APOE4 proteome signature was also unable to differentiate between males and females of the same APOE genotype (APOE4^+^ or APOE4^−^). We also found no differences across APOE genotypes in the CSF levels of ApoE protein or its three isoforms (E2, E3, and E4). This finding was in line with previous studies and a meta‐analysis showing no differences in ApoE protein levels across genotypes (Lehtimaki et al., 1995; Martinez‐Morillo et al., 2014; Talwar et al., 2016). Importantly, we found that our APOE4 protein signature was also independent of AD pathology burden. Using CSF AD biomarkers including Aβ_42_, t‐tau, and p‐tau181 we showed that in nonimpaired healthy controls APOE genotype did not influence the levels of these AD biomarkers. This highlighted that the APOE4 CSF proteome signature in nonimpaired controls was not due to the presence of increased AD pathology burden relative to cognitively normal APOE4 noncarriers. We also found that the APOE4 proteome signature was significantly associated with an increased risk of cognitive decline to MCI or AD over time. To our knowledge, ours is the first study to examine a large number of proteins (6082) and demonstrate that there is a unique APOE4 CSF proteome that is not associated with AD pathology or cognitive status. Our findings are somewhat in line with previous research. A smaller study of less than 300 proteins used linear modeling to show that APOE4 genotype was associated with three unique CSF peptides when controlling for cognitive status (Miles et al., 2021). Further, only a few proteins were shown to be unique to APOE4 carriers when controlling for CSF t‐tau, p‐tau181, and Aβ_42_ (Miles et al., 2021).

Functional enrichment analyses of our identified 57‐protein APOE4 proteome signature indicated significant enrichment for immune responses, inflammation, oxidative and cellular stress response, mitosis dysregulation, DNA damage, catabolism, protein ubiquitination, and synapses. Previous work has similarly highlighted a role for CSF markers of dysregulated immune processes and inflammation in APOE4 carriers. One study showed that APOE4 carriers with AD relative to noncarriers with AD have dysregulated complement pathway proteins that was independent of Aβ_42_ levels (Konijnenberg et al., 2020). Another, on the other hand, linked increased inflammatory cytokines, including IL‐4, IL‐6, and IL‐8, to reduced Aβ deposition and preservation of cognitive function in APOE4 carriers with MCI and early AD (Motta et al., 2020). In a study of cognitively healthy APOE4 carriers, researchers found that APOE4 genotype was associated with decreased CSF TNFα levels (Sasamaya et al., 2020). Importantly, in our study we found that the immune and inflammatory phenotype was reflected in both the brain and the periphery, suggestive of widespread, systemic changes in these processes. This is in line with previous research showing that APOE4 carriers have an increased innate immune response to challenges from lipopolysaccharide (LPS) and toll‐like receptor stimulation (Gale et al., 2014). Further, we found that endothelial cells were significantly enriched for APOE4 proteins, suggestive of BBB dysfunction. A recent study showed that APOE4 carriers have clear BBB breakdown in the hippocampus and medial temporal lobe independent of AD pathology and prior to the onset of cognitive decline (Montagne et al., 2020). This finding is supported by human iPSC‐derived models of the BBB that show endothelial cells have a toxic gain of dysfunction, highlighted by proinflammatory states and cytokine release (Rieker et al., 2019), dysregulated extracellular matrix and endothelial junction integrity (Liu, Zhao, et al., 2022), and cerebral amyloid angiopathy that's driven by calcineurin‐nuclear factor of activated T cells (NFAT) signaling (Blanchard et al., 2020). This idea may support our finding that although more than 50% of our identified proteins were expressed within the hippocampus (26/50 proteins), our proteins were more highly enriched in the caudate and cerebral cortex. It may be the case that our proteins contribute to a BBB breakdown that leads to hippocampal dysfunction characteristic of AD. Future research should continue to use human‐specific models to better understand the role that APOE4, and potential systemic immune dysregulation, plays in driving BBB breakdown and dysfunction.

A search of three molecular interaction databases to examine potential interactions between ApoE and our 57 identified APOE4 CSF proteins (BioGRID v4.4 (Oughtred et al., 2020), UniProt (The Uniprot Consortium, 2022), and IntAct (Del Toro et al., 2022)) yielded few results. Only two of the APOE4 proteins were shown to interact with ApoE itself: forkhead box protein 1 (FOXO1) (Su et al., 2023) and ubiquitin thioesterase ZRANB1 (ZRANB1) (Chen & Zhang, 2022). FOXO1 is a transcription factor involved in insulin signaling and regulation of homeostatic responses to stress and ZRANB1 is a ubiquitin thioesterase involved in Wnt signaling, autophagy regulation, and plays a role in cell morphology and cytoskeletal organization. FOXO1 has been linked to AD. Studies have shown it is involved in the generation of reactive oxygen species (ROS) and the formation of Aβ plaques and tau phosphorylation (Manolopoulos et al., 2010), response to treatment with acetylcholinesterase inhibitors (Paroni et al., 2014), and that high levels of FOXO1 may play a role in AD pathogenesis (Liu, Bai, et al., 2022). To our knowledge, no studies have examined a role of ZRANB1 in AD although there is evidence that targeting ZRANB1 mitigates gliosis and cell loss in retinal neurodegeneration (Wang, Shan, et al., 2018). Future studies should continue to examine the role of FOXO1 and ZRANB1 in APOE4‐associated AD to elucidate their role and determine whether they may be potential treatment targets. It is also important to acknowledge that the levels of CSF ApoE protein and its isoforms in our study did not differ between APOE4 carriers, in line with previous research (Lehtimaki et al., 1995; Martinez‐Morillo et al., 2014; Talwar et al., 2016). This suggests that even though our other identified APOE4‐specific CSF proteins are not currently known to interact with ApoE, or its isoforms, themselves they still may play a role in disease pathogenesis through other, nondirect interaction mechanisms. Mechanistic studies are difficult to undertake in clinical samples and therefore future research would benefit from examining experimentally examining these proteins in human disease‐relevant models. Future research would benefit from examining our identified proteins and enriched cell subtypes as potential precision medicine therapeutic targets for APOE4 carriers at risk of AD.

An important finding of our paper is that the unique APOE4 CSF proteome signature was independent of both AD pathology and cognitive status. Previous studies have yielded mixed results as to whether APOE4 carriers, including those who are cognitively healthy, have higher levels of AD biomarkers indicative of pathology. Some have found that APOE4 carriers have reduced CSF Aβ_42_ (Mehrabian et al., 2015; Morris et al., 2010; Prince et al., 2004; Tapiola et al., 2000; Vemuri et al., 2010), increased t‐tau (Saunders et al., 2022; Tapiola et al., 2000), and increased p‐tau181 (Saunders et al., 2022; Weigand et al., 2023) even when they are cognitively normal. Others have shown that the relationships between APOE4 and CSF levels of Aβ_42_, tau, and p‐tau181 are dependent on age (Kester et al., 2009; Li et al., 2017). In line with this, one multi‐cohort study showed that CSF Aβ42 levels were lower only in older but not younger adults (Lautner et al., 2014). Another study, however, showed that APOE4 was associated with a worsening of age‐related levels of Aβ_42_, p‐tau181, and t‐tau (Toledo et al., 2015). There is also evidence that these effects may be sex‐specific, with another study finding that APOE4 affected CSF t‐tau in both sexes, Aβ_42_ in females but not males, and p‐tau191 in males only (Liu et al., 2021). Another important consideration is differences between one or two APOE4 alleles. Some evidence suggests that healthy controls with one APOE4 allele have unaffected Aβ_42_ CSF levels whereas two APOE4 alleles is associated with a decrease (Hertze et al., 2010).There have also been studies that show that there is no relationship between APOE4 and CSF Aβ_42_ (Engelborghs et al., 2007; Lautner et al., 2014), Aβ_42_/_40_ ratio (Hajjar et al., 2022), tau/Aβ_42_ ratio (Ayton et al., 2017), or tau or p‐tau181 (Engelborghs et al., 2007; Hajjar et al., 2022; Lautner et al., 2014; Mehrabian et al., 2015; Morris et al., 2010; Vemuri et al., 2010). The reasons for these vastly disparate findings across the broader literature are not clear. Therefore, it remains difficult to determine how our finding that APOE genotype was not associated with CSF Aβ42, p‐tau181, and t‐tau fits in with this. This suggests that there is likely a need for a comprehensive meta‐analysis or similar of these studies to better unravel the evidence of associations between APOE genotype and CSF AD biomarkers. Further, without well‐established early predictive indicators of AD, delineating this relationship in clinical populations will likely continue to be difficult. Future studies, therefore, should focus on the use of newer, nonrodent models of AD, such as patient stem cell‐derived models, to shed light on the relationships between APOE4 and AD biomarkers. Another consideration is that in the present study we were unable to examine other AD biomarkers such as Aβ_42/40_ ratio, and p‐tau217 due to a lack of data for included participants. Previous studies of unimpaired controls have found that APOE4 genotype was associated with higher levels of plasma p‐tau217 (Du et al., 2024; Mielke et al., 2022). Another, however, found no effect of APOE genotype on CSF Aβ_42/40_ ratio (Glodzik‐Sobanska et al., 2009). It may be the case that unimpaired healthy controls in our study have the beginnings of AD pathology not captured by our existing biomarkers and warrants follow‐up in future studies.

Our study has limitations. First, the lower n and use of machine learning for proteome profiling precluded examining potential differences between homozygous and heterozygous APOE4 carriers. As outlined in Table 1, there were 61 homozygous carriers in the APOE4^+^ AD group whereas there were 28 and 2 in the APOE4^+^ MCI and APOE4^+^ NI groups, respectively. As documented extensively in the literature, there is a clear relationship between the number of APOE4 alleles and AD risk; with heterozygosity associated with a two to four times increased risk and homozygosity associated with a risk of 15 times or greater risk of AD, although this can vary depending on sex and ethnicity (Belloy et al., 2023; Tsai et al., 1994). Further, there is a growing argument that APOE4 homozygosity may represent a different form of AD that is more closely aligned to autosomal dominant AD (Fortea et al., 2024). Although there may be differences in our APOE4 proteome signature between heterozygous and homozygous carriers, this is unlikely. Our CART models performed strongly and similarly across all performance metrics irrespective of the relative distribution of APOE4 genotypes between the groups. This shows that the presence or absence of homozygous APOE4 patients did not affect our models' ability to use the proteome signature to predict APOE4 carriers, suggesting that all APOE4 carriers have this unique signature. Future research, however, should continue to source larger numbers of homozygous APOE4 carriers to further confirm this finding. A second limitation of our study was an inability to examine males relative to females while preserving their diagnostic categories. This was again due to lower n's and our use of machine learning. The relative distribution of males to females did vary between the groups, ranging from as high as 61% male in the APOE4^−^ AD group to as low as 49% in the APOE4^−^ NI group (Table 1). Our results strongly suggested that the APOE4 CSF proteome status was independent of cognitive status, supporting our experiments where we collapsed males and females across APOE genotype irrespective of diagnosis. Although we showed that our models were unable to differentiate between males and females of the same APOE genotype, future research should look to powering their studies such that sex and diagnosis can be preserved. Sex differences in the effects of APOE4 have been shown previously. For example, female APOE4 carriers with MCI have faster memory decline relative to APOE4 males with MCI (Xiwu et al., 2019), are more likely to progress to further cognitive decline and AD (Altmann et al., 2014), and an interaction between menopause and APOE4 in females that contributes to greater lifetime AD risk (Riedel et al., 2016). There is a suggestion in the literature that inflammation underlies sex differences in AD. Females have been shown to have stronger immune responses and the involvement of different inflammatory pathways and mechanisms (Hanamsagar & Bilbo, 2016; Zhu et al., 2021). Much of the research in this area, however, has focused on microglia, which appear to be differentially regulated in females relative to males likely due to the influence of estrogen (Hanamsagar & Bilbo, 2016; Kodama & Gan, 2019; Lopez‐Lee et al., 2022). In our study, however, although we find that APOE4 is associated with an immune and inflammatory signature, we do not find that microglia are enriched for proteins within this signature. Despite this, it may be the case that microglia in females respond differently to APOE4‐driven inflammation. Future experimental research is needed to better elucidate this relationship.

In conclusion, we found a CSF proteome signature that was unique to APOE4 carriers. This signature was independent of cognitive status, AD pathology as measured by CSF AD biomarkers, and was associated with an increased risk of future cognitive impairment. This suggests that this proteome signature may underly the increased risk of cognitive decline and AD in APOE4 carriers. In addition to being implicated in the BBB, our APOE4 proteins showed enrichment for the immune system and inflammation. From a precision medicine perspective, this suggests that immunomodulation and treatments targeted at improving the BBB may offer the best treatment strategies for APOE4 carriers at risk of AD.

## AUTHOR CONTRIBUTIONS

Concept and design: A.S., C.A.F. Acquisition of data: C.A.F. Statistical analysis: A.S., C.A.F. Interpretation of data: A.S., S.T., A‐N.C., H.M.W., J.H.R., R.H.S., D.A.B., C.A.F. Drafting of the manuscript: C.A.F. Critical review of the manuscript for important intellectual content: A.S., S.T., A‐N.C., H.M.W., J.H.R., R.H.S., D.A.B., C.A.F. Supervision: C.A.F.

## FUNDING INFORMATION

This research did not receive any specific grant from funding agencies in the public, commercial, or not‐for‐profit sectors. A‐N.C. receives funding from the University of Sydney Horizon Fellowship program. H.M.W. receives support from the Alzheimer's Association and the State of Kansas. H.M.W. and R.H.S. are supported by funding from the National Institutes of Health (NIH AG072973). J.H.R. receives funding from the National Health and Medical Research Council (NHMRC), Rebecca Cooper Foundation, and philanthropic support from the Peter Tosi Family. J.H.R. and D.A.B. receive funding from the Medical Research Future Fund (MRFF). C.A.F. receives funding support from Dementia Australia and philanthropic support from the Neil & Norma Hill Foundation, John and Anne Leece Family, Annemarie & Arturo Gandioli‐Fumagalli Foundation, Perpetual Foundation ‐ John Williams Endowment, and Hillcrest Foundation.

## CONFLICT OF INTEREST STATEMENT

None declared.

## CONSENT FOR PUBLICATION

Not applicable.

## Supporting information


Appendix S1.

## Data Availability

Data used in the current study is available through the ADNI database at (https://ida.loni.usc.edu/). New data generated in our analyses are included in the Supplementary Tables.
